# UAV-Assisted Dynamic Monitoring of Wheat Uniformity toward Yield and Biomass Estimation

**DOI:** 10.34133/plantphenomics.0191

**Published:** 2024-06-18

**Authors:** Yandong Yang, Qing Li, Yue Mu, Haitao Li, Hengtong Wang, Seishi Ninomiya, Dong Jiang

**Affiliations:** ^1^ Academy for Advanced Interdisciplinary Studies, Collaborative Innovation Center for Modern Crop Production co-sponsored by Province and Ministry, State Key Laboratory of Crop Genetics and Germplasm Enhancement and Utilization, Nanjing 210095, China.; ^2^ College of Agriculture, National Technique Innovation Center for Regional Wheat Production, Key Laboratory of Crop Ecophysiology, Ministry of Agriculture, Nanjing Agricultural University, Nanjing 210095, China.; ^3^ Graduate School of Agricultural and Life Sciences, The University of Tokyo, Nishi-Tokyo, Tokyo 188-0002, Japan.

## Abstract

Crop uniformity is a comprehensive indicator used to describe crop growth and is important for assessing crop yield and biomass potential. However, there is still a lack of continuous monitoring of uniformity throughout the growing season to explain their effects on yield and biomass. Therefore, this paper proposed a wheat uniformity quantification method based on unmanned aerial vehicle imaging technology to monitor and analyze the dynamic changes in wheat uniformity. The leaf area index (LAI), soil plant analysis development (SPAD), and fractional vegetation cover were estimated from hyperspectral images, while plant height was estimated by a point cloud model from RGB images. Based on these 4 agronomic parameters, a total of 20 uniformity indices covering multiple growing stages were calculated. The changing trends in the uniformity indices were consistent with the results of visual interpretation. The uniformity indices strongly correlated with yield and biomass were selected to construct multiple linear regression models for estimating yield and biomass. The results showed that Pielou’s index of LAI had the strongest correlation with yield and biomass, with correlation coefficients of −0.760 and −0.801, respectively. The accuracies of the yield (coefficient of determination [*R*^2^] = 0.616, root mean square error [RMSE] = 1.189 Mg/ha) and biomass estimation model (*R*^2^ = 0.798, RMSE = 1.952 Mg/ha) using uniformity indices were better than those of the models using the mean values of the 4 agronomic parameters. Therefore, the proposed uniformity monitoring method can be used to effectively evaluate the temporal and spatial variations in wheat uniformity and can provide new insights into the prediction of yield and biomass.

## Introduction

Wheat is an important crop that is widely consumed and cultivated worldwide [1]; 219 million hectares were planted in 2020, accounting for more than 25% of the total cereal crop production [2]. At present, the growth of the world population, the frequent occurrence of extreme weather, and climate change have caused greater demands on wheat [3].

A uniform population structure serves as a critical cornerstone for achieving high crop yields. Populations are composed of individual plants that are interconnected and mutually influenced, engaging in competition for vital resources such as water, nutrients, heat, and light [4–6]. The uneven field topography and disparities in resource distribution resulting from human interventions such as fertilization, sowing, and irrigation, contribute to uneven resource availability among crop individuals. This uneven resource distribution leads to unfavorable competition among crop plants and consequently prevents the formation of a uniform population structure. A uniform population structure in crops confers several advantages, including enhanced light interception [7], reduced light penetration to lower canopy layers, an increased extinction coefficient [8], effective weed suppression [9–11], and uniform resource utilization. The uniform utilization of resources reduces competition among crops. Donald (1968) proposed the concept of ideal plant types (ideotypes) and believed that crops with weak competitors would have high yields [12,13]. Furthermore, the study of uniformity provides a quantitative description of the horizontal structure of populations. Studying the uniformity of plant physiological indicators and environmental factors at the same time can provide valuable information for studying population–environment interactions and explaining spatial distribution patterns [14]. Consequently, timely and accurate monitoring of crop uniformity is highly important. Uniformity serves as both an indicator for selecting superior crop varieties and an indicator of crop growth, guiding breeders and farmers to make informed decisions.

Researchers have proposed various uniformity indices, which can be broadly classified into 3 categories (Table 1). The first category is the traditional index based on the mean or variance [15–19]. These indices are widely applied because of their simple calculations, but they are constrained by plot size and sample size during the sampling process, especially for crops with clustered distributions, where different plot sizes may yield notably different results [14]. The second category is the uniformity index based on distance. These parameters are suitable for studying the uniformity of individual plant spatial distributions with certain spacings [20] but may not be suitable for densely grown crops. The third category is uniformity indices based on entropy or probability. Entropy shares inherent similarities with uniformity, and entropy-based uniformity indices can effectively describe overall uniformity. Entropy has been used as a reference standard for assessing uniformity in some studies [21,22]. However, due to their relatively high computational complexity, these methods are rarely used for high-throughput field crop phenotype analysis.

**Table 1.** Three categories of uniformity indices. In the mean-based index, *S* and $\bar{x}$ represent the standard deviation and mean value, respectively. In the distance-based index, *λ* represents the number of plants within the circle per unit radius; *r* represents the distance from a random plant to its nearest neighbor; *m* represents the number of observation points; *η_j_* represents the CV of 12 plant spacing observations around each observation point relative to the standard plant spacing; *N* is the average number of plants per hole; *a* is the total monopolized sphere; and *A_v_* is the polyhedron volume. In the entropy-based index, *S* is the abundance of one index in the plot, and *P_i_* is the ratio of the number of a certain classification to the total.

Category	Name	Formula	Reference
Mean or variance-based index	Mean crowding	$m^{*}=\left(S^{2}-\bar{x}+{\bar{x}}^{2}\right)/\bar{x}$	[46]
	Clustering index	$I=\left(S^{2}/\bar{x}\right)-1$	[47]
	Coefficient of variation	$\mathrm{CV}=S/\bar{x}$	[48]
Distance-based index	Non-randomness index	$R=2\bar{r}\sqrt{\lambda /\pi }$	[49]
	Uniformity distribution index	$\mathrm{UD}=\frac{1}{\left(1+\sum \;\eta_{j}/m\right)N}$	[39]
	Luo’s index	*L* = *a*/*A_v_*	[23]
Entropy or probability-based index	Shannon entropy	*H*′ = − ∑ *P_i_* ln (*P_i_*)	[50]
	Sheldon’s index	*E_s_* = exp (− ∑ *P_i_* log *P_i_*)/*S*	[51]
	Heip’s index	*E_h_* = [exp(− ∑ *P_i_* log *P_i_*) − 1]/(*S* − 1)	[52]
	Pielou’s index	*J*^′^ = *H*^′^/ ln *S*	[53]
	Alatalo’s index	$\begin{array}{c} E=\left[{\left(\sum P_{i}^{2}\right)}^{-1}-1\right]/\left[\text{exp}(H^{\prime })-1\right] \end{array}$	[54]

Current researches on crop uniformity are not comprehensive, as they predominantly focused on the spatial uniform distribution of individual crops and lacked assessments of uniformity across multiple traits [23]. Canopy uniformity analysis of traits that reflect growth status, including fractional vegetation cover (FVC), leaf area index (LAI), soil plant analysis development (SPAD), and plant height (PH), is also of great significance. Furthermore, traditional methods for measuring traits are performed manually, a process that is labor intensive and inefficient [24]. Unmanned aerial vehicle (UAV)-based crop phenotyping and image processing technology, enabling the high-throughput phenotyping of field crops [25–27], has been widely adopted in the monitoring of LAI, SPAD, PH, and FVC [28–32]. However, there is currently a lack of research to assess the uniformity of the FVC, LAI, SPAD, and PH of crops using UAV-based monitoring.

Current research on uniformity in agricultural crops focuses on the uniformity of the spatial distribution of individual plants [33,34] based on their locations, and is mostly applied to a single growth stage of the crop, such as the seedling stage [35,36]. However, with crop growth, individual plant locations are no longer suitable for evaluating the uniformity of spatial distribution, because crops such as wheat and rice grow very densely and are occluded together. Crop growth in different areas of the same field may vary due to uneven water and fertilizer conditions at this stage. At present, there is no viable method for assessing the uniformity of crop growth at different growing stages. Therefore, a new method is needed to solve the problem that the above effective uniformity assessment methods cannot be applied to intensively grown crops. The innovation of this study is that it not only proposes a uniformity evaluation method applicable to the entire growth stage of wheat but also applies it to the evaluation of wheat growth uniformity.

The objectives of this study were (a) to propose a wheat uniformity assessment method that quantifies wheat uniformity from multiple perspectives, including morphology (LAI, FVC, and PH) and physiology (SPAD); (b) to describe the dynamic changes in wheat uniformity; and (c) to evaluate the effect of uniformity on yield and biomass.

## Materials and Methods

### Field experiment

A 2-year wheat experiment was conducted in 2021 to 2022 (EXP 1) and 2022 to 2023 (EXP 2) at the Baima Experimental Station of Nanjing Agricultural University (N 31°37′08″, E 119°10′28″) in Jiangsu Province (Fig. S1). A total of 210 wheat cultivars (Table S1) with diverse plant architectures, phenological periods, and yield characteristics were selected for this study, each with 3 replicates planted in a total of 630 plots. The plot size was 1.5 × 1.25 m, with a row spacing of 0.25 m and a seeding density of 400 seeds/m^2^. Fertilization was performed according to the following local cultivation practices: nitrogen fertilizer (urea, 240 kg/ha), phosphorus fertilizer (P_2_O_5_, 12%, 120 kg/ha), and potassium chloride (K_2_O, 60%, 120 kg/ha) were applied. For nitrogen fertilizer, a combination of basal and topdressing application was employed, with 50% applied as basal fertilizer before sowing, and the remaining 50% was applied during the tillering stage.

### Data collection and preprocessing

#### Hyperspectral image collection and preprocessing

Hyperspectral images (HSIs) were collected with a Pika-L push broom hyperspectral imaging spectrometer (Resonon, Inc., Bozeman, MT, USA), with a spectral resolution of 2.1 nm between 400 nm and 1,000 nm. It was mounted on a UAV DJI Matrice 600 Pro hexacopter (SZ DJI Technology Co., Ltd., Shenzhen, China), with a global navigation satellite system/inertial measurement unit (GNSS/IMU) system for acquiring georeferenced images and further orthorectification procedures. Drone data were collected at the tillering stage (TS), jointing stage (JS), heading stage (HS), flowering stage (FS), early filling stage (EFS), middle filling stage (MFS), and late filling stage (LFS). The HSIs data were collected at 107, 121, 134, 147, 157, 169, and 183 days after wheat sowing in EXP 1. For EXP 2, the HSIs data were acquired at 109, 131, 145, 155, 166, 178, and 190 days after wheat sowing. The HSIs were collected at a 30-m flight height, with a 50% forward and a 30% lateral overlap, on days with low wind speeds and without obvious cloud cover between 10:00 AM and 2:00 PM local time. A 50% reference plate (Anhui Institute of Optics and Fine Mechanics, Hefei, China) was scanned at the same time for spectral calibration. After HSIs data acquisition, the data were preprocessed with geometric correction using MegaCube 2.11 (Lica United Technology Limited, Beijing, China), georeferencing using ArcMap 10.7 (Esri, Redlands, CA, USA), and image stitching and radiometric calibration using ENVI 5.3 software (Exelis Visual Information Solutions, Boulder, CO, USA). All the HSIs were output with a spatial resolution of 3 cm/pixel.

#### RGB image collection and preprocessing

RGB images were collected with a DJI Phantom 4 UAV (SZ DJI Technology Co., Ltd., Shenzhen, China) with a Sony Exmor R CMOS. The field of view of the camera was 84°. To better estimate the crop height information of wheat, a cross-flight route was adopted. RGB images were collected at a 20-m flight height and with 80% forward and lateral overlap. During the wheat growth stage in which the HSIs were collected, RGB images were taken at 105, 120, 137, 144, 153, 168, and 182 days after wheat sowing in EXP 1. For EXP 2, RGB images were taken at 109, 131, 145, 155, 166, 178, and 190 days after wheat sowing. The flights were conducted under stable sunlight conditions between 10:00 AM and 2:00 PM. The collected RGB images were georeferenced and stitched into orthomosaic and dense point cloud models using Pix4D Mapper software (V4.7.5; Pix4D, Lausanne, Switzerland).

#### Agronomic data collection

The LAI was measured with a SunScan canopy analyzer (Delta-T Devices Ltd., Cambridge, UK), putting the long pole (64 sensors) of the device perpendicular to the wheat planting row. Each plot was measured twice at the one-third and two-thirds locations from the left, and the mean value of these 2 measurements was regarded as the LAI value of the plot. A SPAD meter (SPAD-502 Plus, Konica Minolta, Inc., Osaka, Japan) was used to measure the relative chlorophyll content. During this process, 5 wheat plants were selected using the 5-point sampling method. Then, the SPAD values of the uppermost fully expanded leaves were measured and averaged to represent the SPAD value in each plot. The LAI and SPAD values for all plots were measured twice, at 134 to 135 and 169 to 170 days after wheat sowing in EXP 1. In addition, this study manually measured the height of the tallest plant of all the plots to evaluate the estimated PH. The heights of all plots were measured twice, at 134 and 169 days after wheat sowing in EXP 1.

#### Yield and biomass collection

During the maturation stage, harvesting was conducted manually by late May, and all plants above ground in each plot were harvested. The harvested wheat plants were then gathered in mesh bags for drying, and their biomass and yield were measured and recorded when the moisture content was approximately 12.5%.

Under adverse weather conditions, severe lodging occurred in 21 plots of wheat. Furthermore, some plots experienced partial losses in yield (7 plots) and biomass (10 plots) during harvesting. These anomalous data were subsequently removed during data processing.

### Trait estimation

**Fig. 1.**
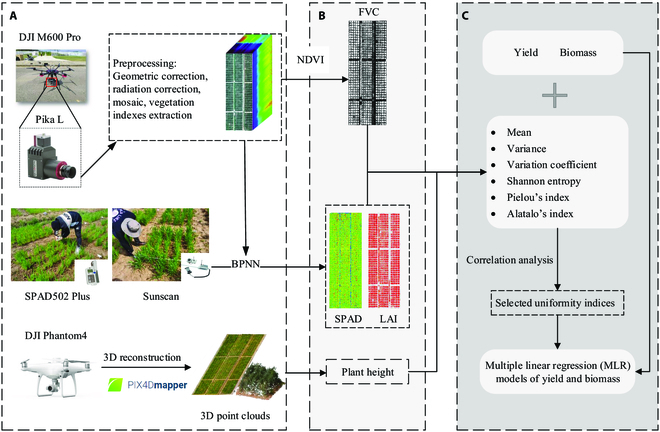
Workflow chart. (A) Data collection and preprocessing. (B) Agronomic parameter estimation. (C) Index selection and modeling.

In this study, 4 traits related to crop growth were estimated from UAV images, including FVC, LAI, and SPAD based on HSIs and PH based on RGB images. The workflow is shown in Fig. 1.

#### FVC extraction based on HSIs

The FVC of the plot was defined as the ratio of the number of vegetation pixels to the total number of pixels in the plot. The normalized difference vegetation index (NDVI) was selected as the parameter for threshold segmentation to extract vegetation pixels since the NDVI is sensitive to the spectral information of vegetation [31]. The 669-nm and 820-nm bands were selected to calculate the NDVI. By extracting valley thresholds from the NDVI grayscale histogram image of 7 stages, the average of these valley values could be used as the threshold (0.44 in this research) for background removal. Then, the FVC of all the plots was calculated, and the crop mask of each stage was generated.

#### Estimation and evaluation of LAI and SPAD based on HSIs

The back propagation neural network (BPNN) is a multilayer feed-forward neural network trained according to the error reverse propagation algorithm, and it is one of the most widely used neural network models [37,38]. This study selected 19 indices (Table S2) and 22 indices (Table S3) as inputs for the LAI and SPAD estimation models, respectively. In this study, the collected LAI and SPAD data were randomly divided into training and test sets at a ratio of 2:1, and the sample sizes of the training and test sets were 840 and 420, respectively. The LAI estimation model has 10 hidden layers, while the SPAD estimation model has 20 hidden layers. Both models have the same number of iterations and learning rate, which are 5,000 and 0.005, respectively. The coefficient of determination (*R*^2^) and root mean square error (RMSE) were used to evaluate the prediction performance for the LAI and SPAD values. MATLAB2019b (MathWorks, Inc., Natick, MA, USA) was selected for model training and testing. Then, the constructed model was used to estimate the LAI and SPAD in EXP2. The output results of the model were visualized and georeferenced, and then the values of all pixels in the plot were extracted from the ROIs.

#### PH estimation based on RGB images

PH was estimated by using the generated 3D dense point clouds. CloudCompare V2.11.3 (www.cloudcompare.org) was used to segment the 3D point cloud of the wheat plants, and the 97th percentile of height was used as the PH of the plot.

#### Uniformity indices

In this study, 4 agronomic parameters (LAI, SPAD, PH, and FVC) were extracted, and their mean, variance, coefficient of variation, Shannon entropy, Pielou’s index, and Alatalo’s index were calculated using the equations provided in Table 1. When calculating entropy-based uniformity indices, 2 input parameters were required: the abundance (number of classifications) and the proportion of each classification. Therefore, a classification parameter (bin width) needed to be set for FVC, LAI, SPAD and PH, and each pixel in the image was assigned a value and then divided into several categories based on the classification parameter. The classification parameters for LAI, SPAD, and PH were 0.5, 2, and 2 cm, respectively. For FVC, each pixel in the plot has only 2 classes of values: 0 or 1. The names of these 20 uniformity indices and the mean values of the 4 agronomic parameters along with their abbreviations are given in Table 2.

**Table 2.** Indices extracted from 4 agronomic parameters

Agronomic parameters	Index name	Abbreviation
FVC	FVC-mean	FM
	FVC-variance	FV
	FVC-coefficient variation	FCV
	FVC-Shannon entropy	FH
	FVC-Pielou’s index	FJ
	FVC-Alatalo’s index	FE
LAI	LAI-mean	LM
	LAI-variance	LV
	LAI-coefficient variation	LCV
	LAI-Shannon entropy	LH
	LAI-Pielou’s index	LJ
	LAI-Alatalo’s index	LE
SPAD	SPAD-mean	SM
	SPAD-variance	SV
	SPAD-coefficient variation	SCV
	SPAD-Shannon entropy	SH
	SPAD-Pielou’s index	SJ
	SPAD-Alatalo’s index	SE
PH	Plant height-mean	PM
	Plant height-variance	PV
	Plant height-coefficient variation	PCV
	Plant height-Shannon entropy	PHH
	Plant height-Pielou’s index	PJ
	Plant height-Alatalo’s index	PE

## Results

### Estimation accuracy of SPAD, LAI, and PH

Table 3 shows the estimation accuracy of the LAI, SPAD, and PH. The BPNN model showed high accuracy for the LAI and SPAD. PH estimated from 3-dimensional dense point clouds also exhibited favorable accuracy, with an *R*^2^ value exceeding 0.80. The precise estimation of agronomic parameters provides a solid foundation for accurately calculating uniformity indices.

**Table 3.** Agronomic parameter estimation accuracy

Agronomic parameters		*R* ^2^	RMSE
SPAD	Training dataset	0.804	3.556
	Test dataset	0.791	3.719
LAI	Training dataset	0.889	0.317
	Test dataset	0.883	0.363
PH		0.812	1.632 cm

### Dynamic changes in wheat uniformity indices

**Fig. 2.**
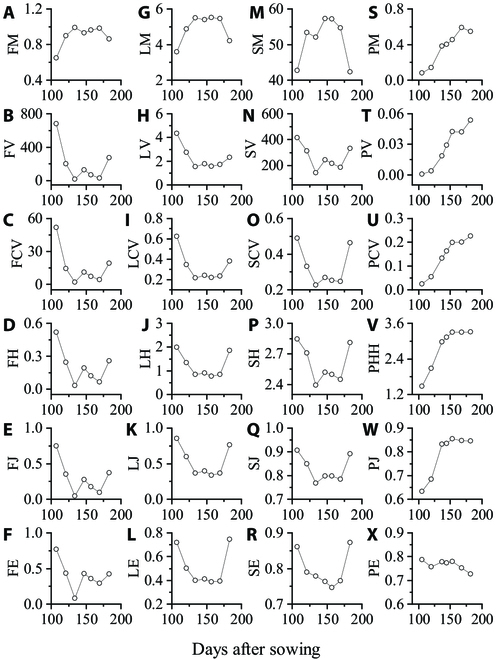
Changes in the wheat uniformity indices throughout the growth stage. (A) to (F) are FM, FV, FCV, FH, FJ, and FE, respectively, extracted from FVC. (G) to (L) are LM, LV, LCV, LH, LJ, and LE, respectively, extracted from the LAI. (M) to (R) are SM, SV, SCV, SH, SJ, and SE extracted from SPAD, respectively. (S) to (X) are PM, PV, PV, PHH, PJ, and PE, respectively, extracted from PH.

The uniformity indices extracted from the same agronomic parameters exhibited similar trends (see Fig. 2). FM (Fig. 2A), LM (Fig. 2G), and SM (Fig. 2M) were negatively correlated with the uniformity indices extracted from the same agronomic parameters. The key growth stages that influenced the uniformity changes were the jointing stage, flowering stage, and late filling stage. The uniformity indices of the 4 agronomic parameters tended to increase before the jointing stage, and the change in the uniformity indices tended to stabilize after the heading stage. Before canopy closure, mixed pixels at the edge of rows caused differences between the parameter values at the row edge and those at the row center, resulting in lower uniformity for FVC, LAI, and SPAD. During the flowering stage, there was a slight decrease in the FVC, LAI, and SPAD uniformity, which may be attributed to drought (Fig. S2), high temperatures, and changes in canopy spectra after flowering. The uniformity slightly increased during the grain-filling stage but decreased again after maturity and senescence. For PH, the uniformity generally rapidly increased first and then tended to stabilize, except for PE, as shown in Fig. 2. The uniformity indices mentioned above were consistent with the visual observations and were able to describe the variations in wheat uniformity to some extent.

### Correlation coefficients between uniformity indices and yield and biomass

**Fig. 3.**
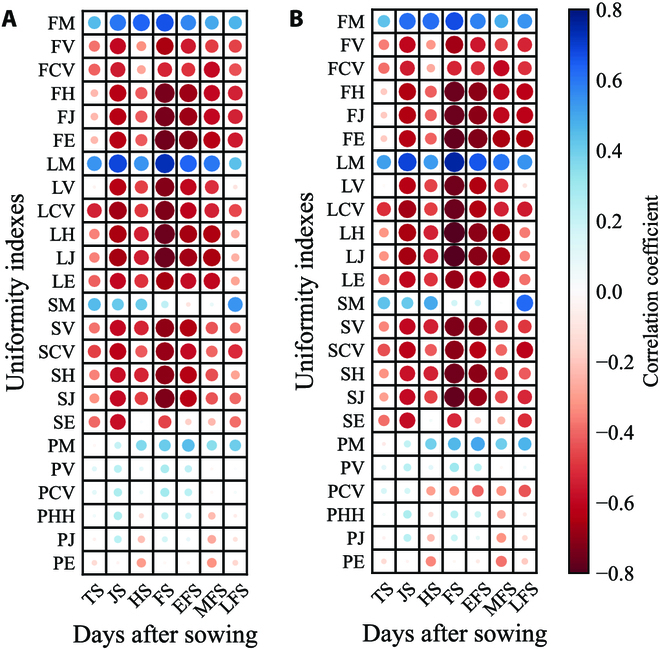
Correlation coefficients between uniformity indices and yield (A) and biomass (B).

Because the uniformity indices change in a similar trend, it is difficult to determine which one is best for use. Therefore, this study conducted a correlation analysis between the uniformity indices and yield and biomass (Fig. 3).

There was a certain regularity in the correlation between the uniformity indices and yield. The uniformity indices extracted from FVC exhibited the strongest correlation with yield during the FS. Among them, FE had the greatest correlation coefficient (*r* = −0.752). For the LAI, before the tillering stage and after senescence, the correlations between each uniformity index and yield were relatively weak, but they remained relatively stable from the jointing stage to the grain filling stage. Among them, LJ had the strongest correlation with yield (*r* = −0.760) at FS. For SPAD, except for the Alatalo index, the maximum correlation coefficients (absolute value) for each uniformity index were also observed at FS. Among them, SJ showed the strongest correlation with yield (*r* = −0.706). Similarly, the mean values of FVC, LAI, and SPAD were positively correlated with yield, whereas the uniformity indices of these 3 parameters were negatively correlated with yield. Compared with those of FVC, SPAD, and LAI, the uniformity indices of PH showed weaker correlations with yield. The strongest correlation between PM and yield was observed for EFS (*r* = 0.447). Among the uniformity indices extracted from the 4 agronomic parameters, LJ at the FS showed the strongest correlation with yield (*r* = −0.760).

The correlation results for final biomass were similar to those for yield. The correlation between the FE index and biomass was strongest (*r* = −0.759) at FS for the FVC. For the LAI, the uniformity indices at FS showed the strongest correlation with the biomass of the LAI, with LJ showing the strongest correlation with biomass (*r* = −0.801). Among the 5 uniformity indices extracted from SPAD, the correlation coefficient (absolute value) between SJ at FS and biomass was the largest (*r* = −0.770). The correlation between the uniformity indices extracted from PH and biomass was generally weak, and the strongest correlation (*r* = 0.508) with biomass was the PM at EFS. In summary, the correlation between LJ at FS and final biomass was the strongest (*r* = −0.801).

### Yield and biomass estimation based on multiparameter indices

**Fig. 4.**
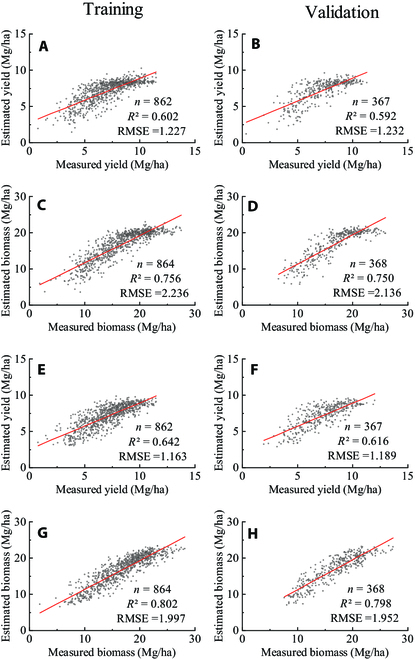
Estimation model of yield and biomass. (A) to (D) are scatter plots of the measured versus estimated values based on the mean values of the agronomic parameters. (E) to (H) are scatter plots for measured versus estimated values based on uniformity indices (FE, LJ, SJ, and PM) of agronomic parameters. The red lines indicate the fitting lines.

Since the data at different times were strongly correlated, we selected the data from 3 key periods—the joining stage (JS), flowering stage (FS), and late filling stage (LFS)—for yield and biomass estimation. We randomly segmented the data into training and validation datasets at a 7:3 ratio and then constructed multiple linear regression (MLR) models based on the uniformity index and mean value, respectively. Finally, the estimated yield and biomass based on the uniformity index and mean value were compared. For models based on uniformity indices, the indices (FE, LJ, SJ, and PM) that showed the strongest correlation with yield and biomass extracted from 4 agronomic parameters (LAI, SPAD, FVC, and PH) were selected as inputs. For models based on mean values, the mean values (FM, LM, SM, and PM) of the 4 agronomic parameters were also selected to construct an MLR model for comparison (Fig. 4). The validation results showed that the accuracies of the yield model (*R*^2^ = 0.616, RMSE = 1.189 Mg/ha) and biomass model (*R*^2^ = 0.798, RMSE = 1.952 Mg/ha) based on the uniformity indices were better than those of the models constructed using the mean values of the 4 agronomic parameters. The accuracy of the uniformity index-based models was also better than that of the mean-based models in the training dataset. The model formulas were as follows:

$$\begin{array}{c} y_{\text{yiel}d\_mean}=4.421{\mathrm{FM}}_{\mathrm{JS}}-3.915{\mathrm{FM}}_{\mathrm{FS}}+2.537{\mathrm{FM}}_{\mathrm{LFS}}+1.081{\mathrm{LM}}_{\mathrm{JS}}\\ \;+1.808{\mathrm{LM}}_{\mathrm{FS}}+0.399{\mathrm{LM}}_{L\mathrm{FS}}-0.067{\mathrm{SM}}_{\mathrm{JS}}-0.058{\mathrm{SM}}_{\mathrm{FS}}\\ \;-0.025{\mathrm{SM}}_{LFS}+0.297{\mathrm{PM}}_{\mathrm{JS}}-2.012{\mathrm{PM}}_{\mathrm{FS}}+1.681{\mathrm{PM}}_{\mathrm{LFS}}-4.934 \end{array}$$ (1)

$$\begin{array}{c} y_{\text{biomass}\_\text{mean}}=8.575{\mathrm{FM}}_{\mathrm{JS}}-8.184{\mathrm{FM}}_{\mathrm{FS}}+4.411{\mathrm{FM}}_{\mathrm{LFS}}\\ \;+2.594{\mathrm{LM}}_{\mathrm{JS}}+3.819{\mathrm{LM}}_{\mathrm{FS}}+1.195{\mathrm{LM}}_{\mathrm{LFS}}-0.127{\mathrm{SM}}_{\mathrm{JS}}\\ \;-0.182{\mathrm{SM}}_{\mathrm{FS}}-0.063{\mathrm{SM}}_{\mathrm{LFS}}-2.572{\mathrm{PM}}_{\mathrm{JS}}+2.145{\mathrm{PM}}_{\mathrm{FS}}\\ \;+0.371{\mathrm{PM}}_{\mathrm{LFS}}-8.566 \end{array}$$ (2)

$$\begin{array}{c} y_{\text{yield}\_\text{index}}=6.621-0.290{\mathrm{FE}}_{\mathrm{JS}}-1.193{\mathrm{FE}}_{\mathrm{FS}}\\ \;-1.561{\mathrm{FE}}_{\mathrm{LFS}}-1.960{\mathrm{LJ}}_{\mathrm{JS}}-2.559{\mathrm{LJ}}_{\mathrm{FS}}\\ \;-1.662{\mathrm{LJ}}_{\mathrm{LFS}}+3.085{\mathrm{SJ}}_{\mathrm{JS}}-2.517{\mathrm{SJ}}_{\mathrm{FS}}\\ \;+4.700{\mathrm{SJ}}_{\mathrm{LFS}}-0.038{\mathrm{PM}}_{\mathrm{JS}}-1.418{\mathrm{PM}}_{\mathrm{FS}}\\ \;+1.411{\mathrm{PM}}_{\mathrm{LFS}} \end{array}$$ (3)

$$\begin{array}{c} y_{\text{biomass}\_\text{index}}=13.609-0.261{\mathrm{FE}}_{\mathrm{JS}}-0.688{\mathrm{FE}}_{\mathrm{FS}}\\ \;-3.295{\mathrm{FE}}_{\mathrm{LFS}}-7.823{\mathrm{LJ}}_{\mathrm{JS}}-7.212{\mathrm{LJ}}_{\mathrm{FS}}\\ \;-3.282{\mathrm{LJ}}_{\mathrm{LFS}}+13.550{\mathrm{SJ}}_{\mathrm{JS}}+0.654{\mathrm{SJ}}_{\mathrm{FS}}\\ \;+0.125{\mathrm{SJ}}_{\mathrm{LFS}}-2.570{\mathrm{PM}}_{\mathrm{JS}}+1.866{\mathrm{PM}}_{\mathrm{FS}}\\ \;+0.605{\mathrm{PM}}_{\mathrm{LFS}} \end{array}$$ (4)

where *y_yield_mean_* and *y_biomass_mean_* represent the yield and biomass based on the means, respectively; *y_yield_index_* and *y_biomass_index_* represent the yield and biomass based on the indices, respectively; and JS, FS, and LFS represent the joining stage, flowering stage, and late filling stage, respectively, which are the stages of agronomic data collection; please refer to Table 2 for FM, LM, SM, PM, FE, LJ, and SJ.

## Discussion

This study describes a high-throughput quantitative approach for determining the multiparameter uniformity of wheat throughout the entire growth stage. In this study, we designed a trial with 210 wheat cultivars and collected high-resolution hyperspectral and RGB data using drones. Subsequently, 4 agronomic parameters, LAI, SPAD, PH, and FVC, were estimated from the UAV data, from which 20 uniformity indices were extracted. The dynamics of uniformity and its impact on yield and final biomass were then analyzed. However, different wheat cultivars, UAV data collection methods, and classification parameter settings all have a direct impact on wheat uniformity; therefore, it is necessary to discuss their effects.

### Differences in uniformity among wheat cultivars

**Fig. 5.**
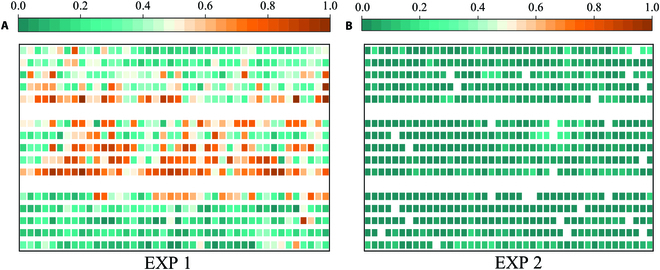
LJ values of wheat at 147 days after sowing in EXP 1 (A) and 155 days after sowing in EXP 2 (B). Each color block corresponds to a plot in the field trial, and the white regions are plots with abnormal growth.

Since this study selected a large number of cultivars with different plant structures, phenological stages, and yield characteristics, and because uniformity is related to yield [39], the difference in uniformity (LJ at FS) among cultivars was analyzed by 2-way analysis of variance (ANOVA, 2 years—210 cultivars), which revealed a significant difference in uniformity among the cultivars (*P* < 0.001). The differences among cultivars suggest that LJ is genetically determined, which needs to be further studied in the future. In addition, the growth of wheat in different years may be very different due to the influence of climate. The LJ indices of EXP 1 and EXP 2 are presented in Fig. 5, showing that the growth of EXP 2 was more uniform than that of EXP 1. The results of the above ANOVA also showed a significant difference in uniformity between years (*P* < 0.001). This is because there was no artificial irrigation in either year of the experiment, the climate was hot and dry with little rainfall in EXP1 during the critical growth stages, and the climate was favorable with abundant precipitation in EXP2 (Fig. S2). Drought affects crop metabolism, development, and habits, resulting in reduced leaf area and yield loss in the field [40].

**Fig. 6.**
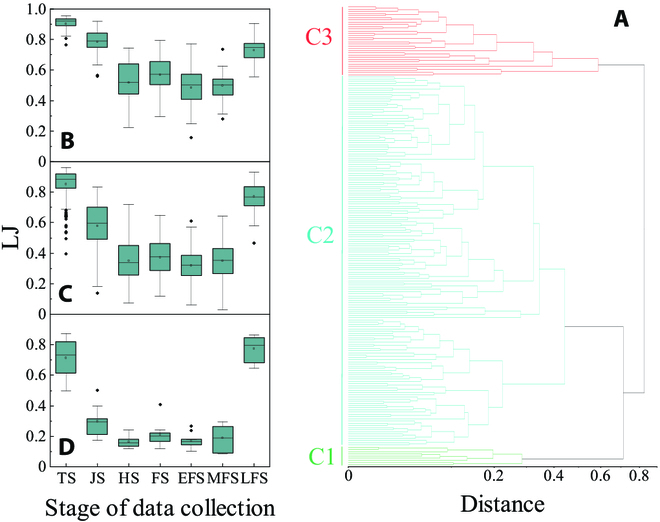
Wheat cultivar hierarchical cluster analysis. (A) Hierarchical cluster analysis of 210 wheat cultivars based on yield and biomass. (B) to (D) show the dynamic changes in the wheat LJ indices of C3, C2, and C1, respectively.

To further examine the relationships among yield, biomass and uniformity, this study conducted hierarchical cluster analysis. According to the measured yield and biomass, all cultivars were divided into 3 categories: C1, C2, and C3, as shown in Fig. 6A. ANOVA was conducted for the yield and final biomass of the wheat varieties within the 3 categories. The results indicated significant differences in yield and final biomass among the 3 categories (Table 4), confirming the effectiveness of this classification method. Figure 6B to D illustrate the dynamic changes in the LJ uniformity indices for these 3 categories of cultivars. The uniformity was positively correlated with the yield. In other words, wheat cultivars with higher yields or biomass have stable and well-structured populations, resulting in smaller differences in growth among wheat populations and thus leading to a more uniform wheat growth, which is consistent with previous research results [9,41].

**Table 4.** Mean values and *P* values of the 3 categories of wheat cultivars. A *P* value <0.05 indicates a significant difference.

	Yield (kg/hm^2^)	Biomass (kg/hm^2^)	LJ
C1	8,605.4	18,207.2	0.212
C2	6,719.1	15,017.6	0.372
C3	4,932.0	11,262.6	0.569
*P* value	1.08 × 10^−37^	5.60 × 10^−44^	9.70 × 10^−15^

### Effect of the classification parameter on the entropy-based uniformity indices

Entropy is widely used in many disciplines [42,43] and can serve as a reference for describing uniformity in certain applications. In ecology, entropy is also used to calculate indices that describe the uniformity among species in an ecological community, such as Pielou’s index. Calculating the number and proportion of a species in ecology is a straightforward process [43]. However, this method is not applicable to agriculture. Therefore, we introduced a classification parameter (bin width) to categorize the values of agronomic parameters into several classes. When the classification parameter is sufficiently large, all values are grouped into one class, resulting in an entropy value of 0 and rendering the result meaningless. Conversely, if the classification parameter is too small, it results in unnecessary calculations, as any 2 distinct values will be placed in separate classes. Therefore, it is necessary to determine an appropriate classification parameter for uniformity indices that incorporate entropy.

**Fig. 7.**
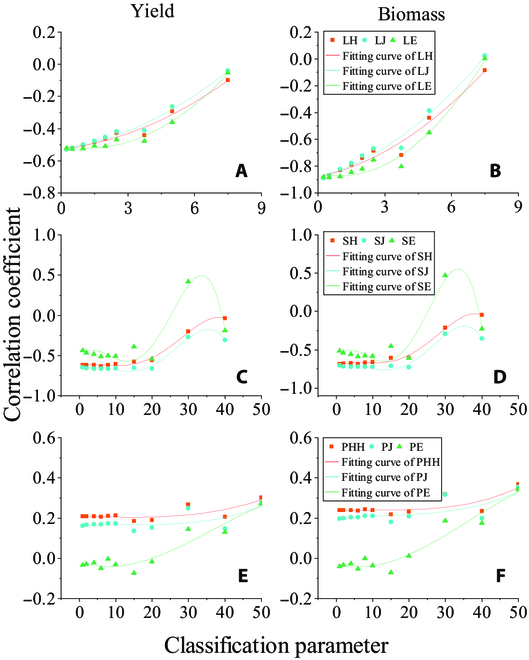
Effect of classification parameters on entropy-based uniformity indices. (A and B) Correlations between the LAI uniformity indices obtained with different classification parameters and yield and biomass. (C and D) Correlations between the SPAD uniformity index obtained by different classification parameters and yield and biomass. (E and F) Correlations between the PH uniformity indices obtained with different classification parameters and yield and biomass.

For a given growth stage of wheat, the abundance and probability of each classification can be determined by defining classification parameters. This study conducted an analysis of the classification parameters for the LAI, SPAD, and PH. Nine classification parameters were set for the LAI: 0.25, 0.5, 1, 1.5, 2, 2.5, 3.75, 5, and 7.5; 10 classification parameters were set for the SPAD: 1, 2, 4, 6, 8, 10, 15, 20, 30, and 40; and 11 classification parameters were set for the PH: 1, 2, 4, 6, 8, 10, 15, 20, 30, 40, and 50 (cm). Subsequently, Shannon entropy, Pielou’s index, and Alatalo’s index were computed based on each of the classification parameters of the LAI, SPAD, and PH, and correlation analyses were performed with yield and biomass, as shown in Fig. 7. The correlation between the 3 uniformity indices and yield and biomass increased as the classification parameter decreased for LAI. The growth rate of the correlation coefficient stabilized when the LAI classification parameter was less than 1. For SPAD, the correlation coefficients of the 3 uniformity indices varied less when the classification parameter was less than 20. The correlations of the 3 uniformity indices with yield and biomass for PH were relatively low, indicating that changes in the classification parameters had little effect on the correlation coefficients.

### Effect of spatial resolution on uniformity indices

**Fig. 8.**
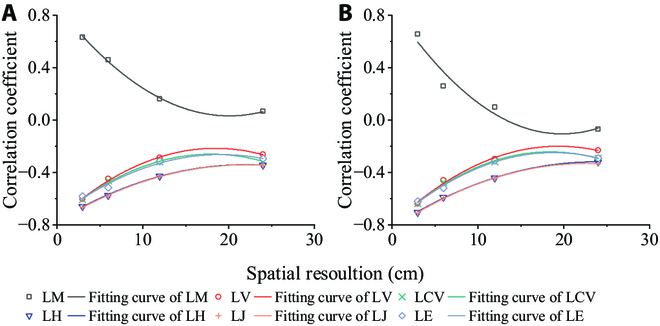
Effect of spatial resolution on uniformity indices. (A) The correlation coefficient between the uniformity indices and yield. (B) The correlation coefficients between the uniformity indices and biomass.

Spatial resolution is an indicator used to characterize the level of detail and differentiation of ground targets in an image, which has a certain impact on the extraction of agronomic parameters in experimental fields [44]. Although the images captured by UAV platforms already have a higher spatial resolution than satellite platforms, the short endurance time of UAV platforms often leads researchers to increase the flight altitude while maintaining a certain resolution. This results in a larger ground sampling distance (GSD) and an increased number of mixed pixels in the image, leading to the loss of fine image information [45]. Therefore, if the wheat canopy is not completely closed, a larger GSD will result in more mixed pixels between the background and the wheat canopy, thereby affecting the accuracy of the data. To analyze the impact of spatial resolution on the results, we collected wheat HSIs at 4 resolutions with GSDs of 3 cm, 6 cm, 12 cm, and 24 cm. The uniformity index was extracted using the same method and stratification parameters, and correlation analysis was conducted between yield and biomass (Fig. 8). The analysis results showed that the correlation coefficients between all uniformity indices and yield increased with increasing resolution. Due to hardware limitations, the HSIs collected in this experiment reached the highest spatial resolution (3 cm). However, from the line graph, it can be observed that there is no saturation point at the highest spatial resolution, indicating that further increasing the spatial resolution can improve the accuracy of the uniformity index.

### Future perspectives

Different uniformity indices may have different applications. The uniformity indices derived from the 4 agronomic parameters are not interchangeable because they represent different aspects of wheat uniformity. The PH uniformity index mainly describes the vertical uniformity of the canopy structure, while the coverage uniformity index describes the horizontal uniformity of the structure. The SPAD uniformity index represents the physiological uniformity of wheat, while the LAI uniformity index represents the morphological uniformity. The integration of these uniformity indices improved the estimation of yield and biomass compared with that using mean values as shown in Fig. 4, which shows great potential for use as the input parameters of yield and biomass estimation models in future research. In addition, uniformity can also be used to select sample areas for yield estimation in fields, because the analysis results of this study show that yield and uniformity are positively correlated. On the other hand, there was a significant difference in uniformity among cultivars, suggesting that uniformity may become one of the selection criteria for productivity in future wheat breeding. To verify this, the relationship between uniformity and productivity at each growth stage and the comparison of the heritability of uniformity at each growth stage should be investigated in the future.

Furthermore, this study focused only on the analysis of wheat. Further data analysis and validation are required for other crops.

## Data Availability

The datasets analyzed in this study are available from the corresponding authors upon reasonable request.
