# Supplementary figures and images for: UAV-Assisted Dynamic Monitoring of Wheat Uniformity toward Yield and Biomass Estimation
Source: Plant Phenomics. 2024 Jun 18;6:0191. doi: 10.34133/plantphenomics.0191 (PMC11184949; doi:10.34133/plantphenomics.0191)

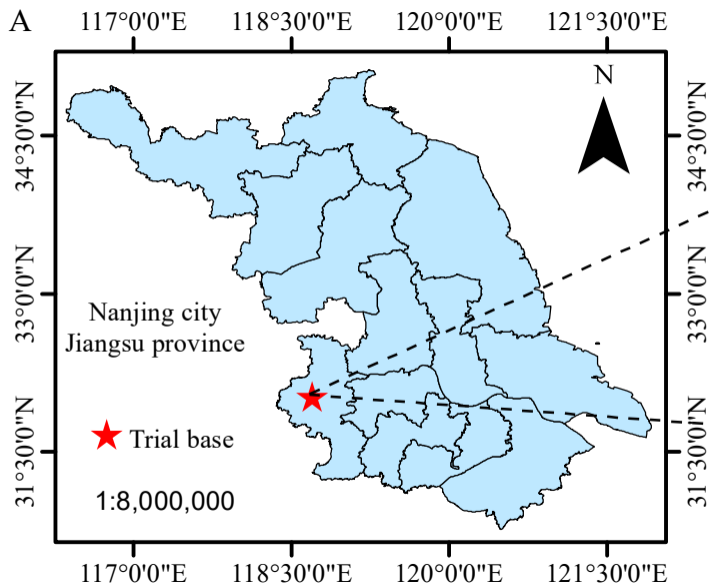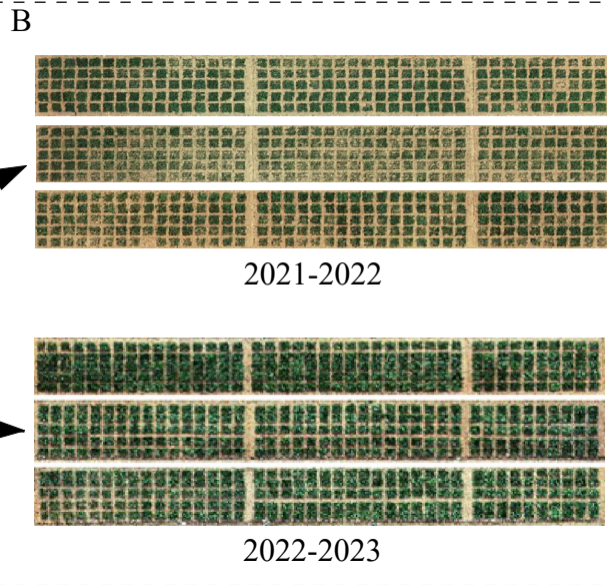

Supplement: Supplementary 1 — Figs. S1 and S2 Tables S1 to S3 [file plantphenomics.0191.f1.zip › Fig.S1.pdf]

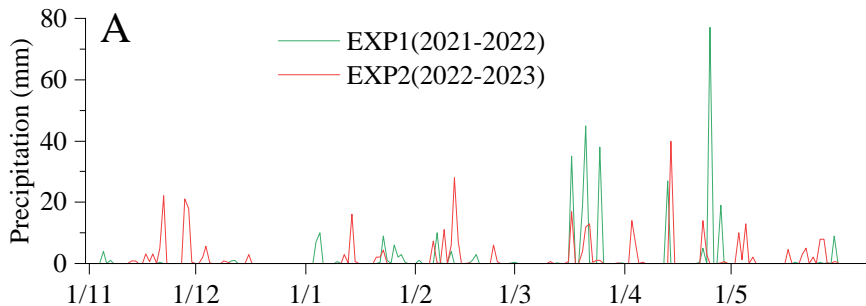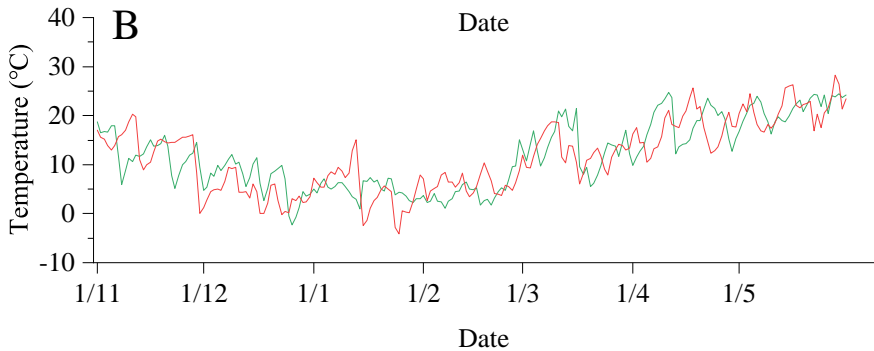

Supplement: Supplementary 1 — Figs. S1 and S2 Tables S1 to S3 [file plantphenomics.0191.f1.zip › Fig.S2.pdf]
